# The value of metagenomic next-generation sequencing in lower respiratory tract infections among critically ill patients in the ICU

**DOI:** 10.3389/fcimb.2026.1746117

**Published:** 2026-02-25

**Authors:** Ying Xu, Weiwei Wu, Danjiang Dong, Ning Liu, Jia Liu, Baocui Qi, Qin Gu

**Affiliations:** 1 Department of Intensive Care Medicine, Nanjing Drum Tower Hospital, Affiliated Hospital of Medical School, Nanjing University, Nanjing, China; 2 China Hospital Reform and Development Research Institute of Nanjing University, Nanjing Drum Tower Hospital, Nanjing, China; 3 Department of Medicine, Dinfectome Inc., Nanjing, Jiangsu, China

**Keywords:** conventional microbiological testing, intensive care unit, lower respiratory tract infections, metagenomic next-generation sequencing, pathogen detection

## Abstract

Lower respiratory tract infections (LRTIs) frequently occur as a severe complication in intensive care unit (ICU) patients, substantially raising patient mortality rates and extending hospitalization periods. In this study, a retrospective cohort study of 261 suspected LRTI patients in the ICU of Nanjing Drum Tower Hospital between April 2021 and February 2024 was conducted. The results showed that metagenomic next-generation sequencing (mNGS) had a sensitivity of 80.1%, a specificity of 35%, and an accuracy of 66.3% across all samples. For pathogen detection, mNGS outperformed conventional microbiological testing (CMT) in detecting bacteria and DNA viruses, while CMT had a slight advantage in RNA virus detection, though the difference was not statistically significant (*p* = 0.305). When comparing microbial profiles between survival and death groups, survivors had a more diverse pathogen spectrum, particularly in bacteria and RNA viruses. There were 262 species detected in both groups, with *Corynebacterium striatum* being the dominant species in the survival group and *Pseudomonas aeruginosa* in the death group. In the 128 patients whose treatment plans were adjusted based on mNGS results, 59.4% underwent escalation, 25.8% had their medications changed, and 1.6% initiated new treatment regimens. Further follow-up revealed that mNGS - guided treatment adjustments were effective in improving clinical symptoms in 58.6% of ICU patients. A predictive model for patient outcomes was developed utilizing the random forest algorithm, achieving an area under the receiver operating characteristic curve (AUC) of 0.722.

## Introduction

Lower respiratory tract infections (LRTIs) are a frequent and severe complication among critically ill patients in the intensive care unit (ICU), significantly increasing mortality rates, prolonging hospital stays, and exacerbating the medical burden (Feldman and Shaddock, 2019; Oliveira et al., 2023). In these patients, compromised immune function, extensive use of mechanical ventilation, and a higher frequency of invasive procedures collectively contribute to a more complex and diverse etiology of LRTIs. Accurate and rapid pathogen identification is crucial for the rational use of antimicrobial agents and for improving patient outcomes (Charlton et al., 2018; Moreau et al., 2018; Liu et al., 2022).

Traditional pathogen detection methods, such as culture, microscopy, and polymerase chain reaction (PCR), are widely used in clinical practice but have limitations in sensitivity and specificity, particularly when dealing with complex or mixed infections. In recent years, metagenomic next-generation sequencing (mNGS) has emerged as a powerful tool for diagnosing LRTIs due to its high-throughput and unbiased pathogen detection capabilities. Compared with conventional methods, mNGS offers higher sensitivity, enabling the identification of low-abundance pathogens that are critical for early diagnosis (He et al., 2022a). Additionally, the rapid turnaround time of mNGS, with results available within 24 hours, enables timely clinical decision-making and facilitates prompt adjustments to treatment plans (Lv et al., 2024).

Moreover, mNGS has demonstrated unique value in diagnosing LRTIs in critically ill patients in ICU. Multiple studies have shown that mNGS can detect pathogens missed by traditional methods, thereby altering treatment strategies for some patients (Benoit et al., 2024; Lv et al., 2024). However, despite its superior performance in pathogen detection, the application of mNGS in critically ill ICU patients faces several challenges, including high costs, diverse sample processing requirements, complex result interpretation, and the potential for false positives or false negatives (Filkins et al., 2020; Miller and Chiu, 2022). Nonetheless, as the technology continues to advance and improve, mNGS is poised to become an essential tool for LRTI diagnosis in critically ill ICU patients. This study aims to evaluate the clinical utility of mNGS in ICU patients with LRTIs, focusing on its role in enhancing diagnostic accuracy, guiding antimicrobial therapy, and improving patient outcomes.

## Methods

### Patient characteristics

**Figure 1:**
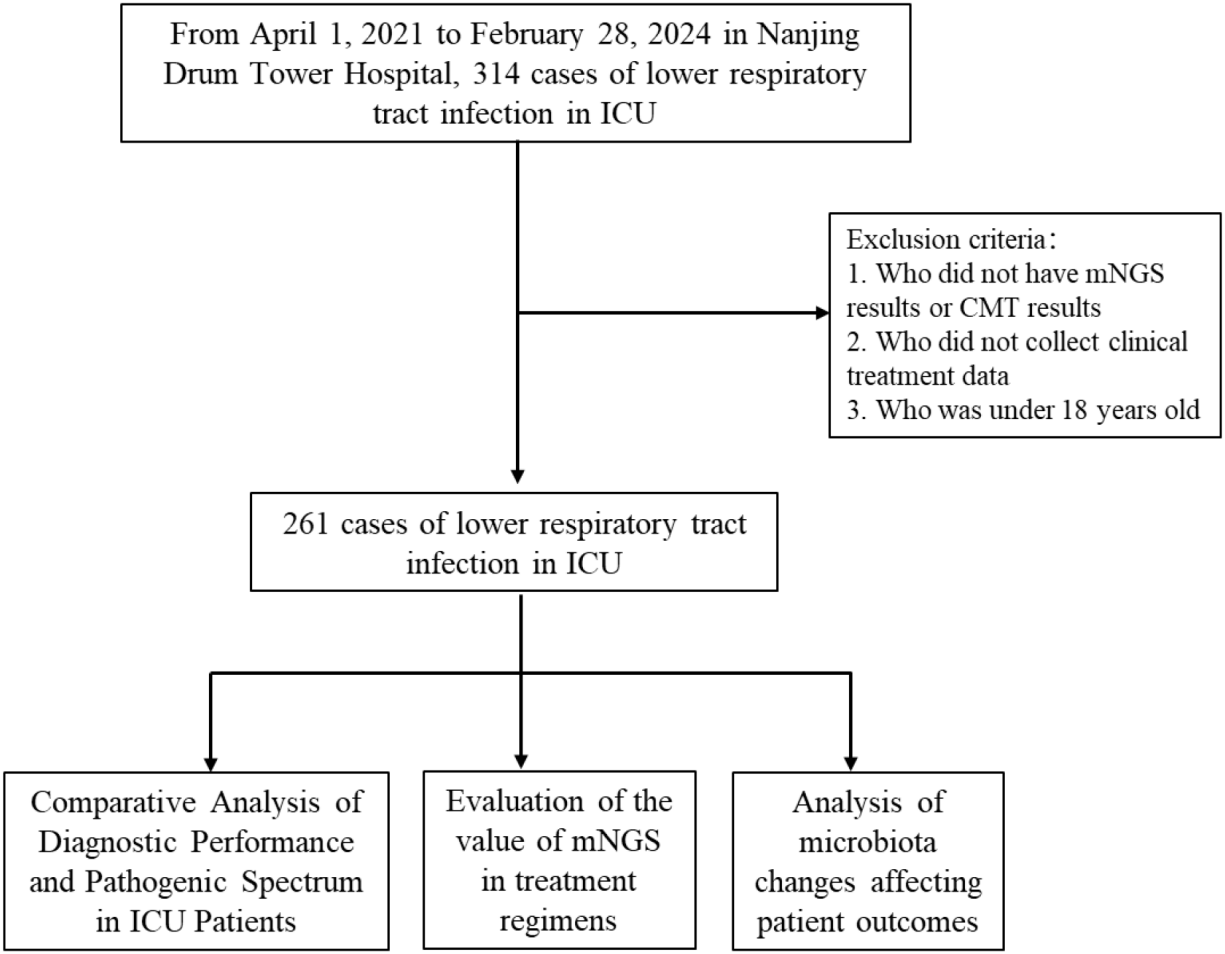
Overview of patient enrollment workflow.

We conducted a retrospective cohort study of 261 patients with suspected LRTIs from the ICU of Nanjing Drum Tower Hospital, China, between April 2021 and February 2024. The inclusion criteria were as follows: 1) patients admitted to the ICU for ≥24 hours; 2) patients who met the diagnostic criteria for LRTIs as defined in the Diagnostic Criteria for Hospital Infections (Trial), which included clinical symptoms such as fever, cough, abnormal sputum production, and dyspnea, as well as physical examination findings such as wet rales on lung auscultation and radiological evidence of lung abnormalities; 3) patients aged ≥18 years; 4) Lower respiratory tract samples of sufficient quality for mNGS analysis; and 5) patients with complete clinical treatment records available for review. The exclusion criteria were as follows: 1) patients had an ICU stay of less than 24 hours; 2) patients who lacked complete clinical data necessary for analysis, including microbiological testing results and treatment records; 3) patients aged <18 years. The study workflow was illustrated in **Figure 1**. Ethical approval was obtained from the Ethics Committee of Nanjing Drum Tower Hospital (No. 2023-061-01). Written informed consent was obtained from patients or their legally authorized representatives prior to sample collection.

### Collection of samples

A total of 261 samples were collected for concurrent conventional microbiological testing (CMT) and mNGS analysis, including 119 samples of alveolar lavage fluid (BALF), 105 samples of blood, 30 samples of cerebrospinal fluid, and 7 samples of other types. For BALF collection, patients received local anesthesia of the throat, followed by the introduction of a fiberoptic bronchoscope to irrigate the lesion site multiple times. The fluid was then adequately suctioned and collected in a sterile container. Blood samples were collected using cfDNA anticoagulant tubes under negative pressure. Pleural fluid samples were obtained by clinicians via percutaneous puncture or surgical approaches under strict aseptic conditions. The puncture site was localized by imaging or percussion, sterilized, and anesthetized, followed by extraction of the pleural fluid using a hollow-bore needle. After collection, all samples were sent to Dinfectome Inc. for mNGS testing, while conventional pathogen testing (including bacterial culture, viral serology, G test and GM test for fungi, and acid-fast staining for tuberculosis, etc.) was performed in the hospital laboratory.

### Clinical data collection

Demographic data, laboratory test results, therapeutic interventions, and clinical outcomes were extracted from patient electronic medical records via the hospital’s integrated information system. Additionally, information regarding pre-admission antibiotic therapy, initial antibiotic regimens upon admission, and subsequent modifications based on mNGS results was also collected. The clinical impact of mNGS on management was adjudicated by medical record review and categorized as positive, negative, no effect, or indeterminate. Positive impact referred to clinically compatible and actionable mNGS findings that supported diagnosis and informed antimicrobial management, including escalation, de-escalation, replacement, enablement, or maintenance; negative impact referred to clinically incompatible findings that led to potentially inappropriate management changes, whereas no effect indicated unchanged management and indeterminate indicated that the contribution of mNGS could not be reliably assessed due to insufficient documentation or confounding factors.

### mNGS test

#### Nucleic acid extraction, library preparation and sequencing

Plasma was prepared from blood samples and circulating cell-free DNA (cfDNA) was isolated from plasma with the QIAamp Circulating Nucleic Acid Kit (Qiagen) according to the manufacturer’s protocols. BALF samples were transferred to a Lysing Matrix tube and mechanically lysed prior to extraction. DNA from other specimen types was extracted using the TIANamp Magnetic DNA Kit (Tiangen) according to the manufacturer’s protocols. In addition, total RNA was extracted from specimens using the QIAamp Viral RNA Mini Kit (Qiagen), followed by rRNA depletion to enable RNA virus detection. In parallel, no-template controls (NTCs) consisting of UltraPure DNase/RNase-free distilled water were processed using the same extraction workflow. Sequencing libraries were constructed using the Hieff NGS C130P2 OnePot II DNA Library Prep Kit for MGI (Yeasen Biotechnology) according to the manufacturer’s instructions, and library quality was assessed on an Agilent 2100 Bioanalyzer. Libraries were sequenced as 50-bp single-end reads on the DIFSEQ-200 platform, generating approximately 20 million reads per sample.

### Bioinformatics analysis

Raw sequencing data were split by bcl2fastq2 (version 2.20), and high-quality sequencing data were generated using Trimmomatic (version 0.36) by removing low-quality reads, adapter contamination, duplicated and shot reads (length<36 bp). Human host sequences were subtracted by mapping to the human reference genome (hs37d5) using bowtie2 (version 2.2.6), and the proportion of human reads was calculated for each sample as a sequencing quality metric. Reads that could not be mapped to the human genome were retained and aligned to the microorganism genome database for microbial identification by Kraken (version 2.0.7), and for species abundance estimation by Bracken (version 2.5.0). The microorganism genome database contained genomes or scaffolds of bacteria, fungi, viruses and parasites (downloaded from GenBank release 238, ftp://ftp.ncbi.nlm.nih.gov/genomes/genbank/).

### Interpretation and reporting

The mNGS pathogen detection pipeline was described in previous studies (Xu et al., 2023). The criteria for defining a positive detection were as follows: (1) At least one species-specific read was required for the detection of Mycobacterium, Nocardia, and Legionella pneumophila; (2) For other bacteria, fungi, viruses, and parasites, a minimum of three unique reads was required; (3) Pathogens were excluded if the ratio of microorganism reads per million of a given sample compared to the NTC was less than 10. Organisms meeting the above thresholds were subsequently interpreted for clinical relevance by integrating specimen type and clinical context. Briefly, detections were considered likely pathogens when they were concordant with the patient’s symptoms or supported by clinical microbiological test results, whereas organisms consistent with typical oral flora in the absence of supportive clinical evidence were interpreted as commensals.

### Microbial flora analysis

Alpha diversity was estimated by the Shannon index based on the taxonomic profile of each sample, beta diversity was assessed by the Bray-Curtis measure, and compared between ICU patients by using Wilcoxon rank sum test, and was subsequently visualized by principal coordinate analysis (PCoA) plot. PERMANOVA was performed by the R package “vegan” to analyze Bray-Curtis distance in different survival and death groups. Differential relative abundance of taxonomic groups at the genus level among groups was tested by using Kruskal-Wallis rank sum test. To account for multiple comparisons across taxa, p-values from differential abundance testing were adjusted using the Benjamini–Hochberg false discovery rate (FDR) procedure, with FDR-adjusted *p* < 0.05 considered statistically significant. The species with mean relative abundances greater than 0.1% and penetrance greater than 10% among all samples were compared. Statistically significant differences in the relative microbial abundance among groups were assessed by the linear discriminant analysis of effect size (LEfSe) analysis.

### Construction of a predictive model based on machine learning

A diagnostic prediction model for clinical outcomes in critically ill patients was developed by integrating the abundances of significantly differential microbial species with key clinical variables as predictive features. To mitigate the randomness inherent in conventional train-test splits given the limited sample size, a rigorous leave-one-out cross-validation (LOOCV) framework was adopted as the core strategy for model training and evaluation. In each LOOCV iteration, a single sample was held out as the independent test set, while all remaining samples constituted the training set, ensuring that every sample was predicted independently once. Using the H2O machine-learning platform, four distinct types of prediction models were constructed and evaluated in parallel: Gradient Boosting Machine (GBM), Generalized Linear Model with elastic-net regularization (GLM), Random Forest (RF), and Deep Learning neural network (DL). During the training of each model, an additional 10-fold cross-validation was implemented on the LOOCV training set to guide hyperparameter tuning and mitigate overfitting, thereby enhancing generalizability. Model performance was primarily assessed using receiver operating characteristic (ROC) curves and the area under the curve (AUC). The 95% confidence intervals for the AUC were calculated using DeLong’s method, and the optimal classification threshold was determined by maximizing the Youden index, from which sensitivity, specificity, and other diagnostic metrics were derived. Predicted probabilities, ROC curve coordinates, and all performance metrics for each model were systematically recorded for comprehensive comparison. The model that demonstrated the highest AUC alongside robust diagnostic performance under the LOOCV framework was selected as the final predictive tool.

### Treatment response assessment

Treatment outcomes were categorized as clinical improvement, deterioration, no change, death, or untraceable according to the Chinese guidelines for the diagnosis and treatment of adult community-acquired pneumonia and medical record review. Clinical improvement was defined as marked resolution of infection, evidenced by normalization of infection-related markers (e.g., C-reactive protein and white blood cell count), radiological improvement on chest CT (reduction or resolution of lesions), and substantial relief of respiratory symptoms (decreased cough frequency, sputum changing from purulent to thinner, resolution of chest tightness, and alleviation of chest pain). Deterioration was defined as infection-related clinical worsening, reflected by worsening symptoms, uncontrolled infection markers, or radiological progression. No change was defined as persistent symptoms with no meaningful improvement in infection markers or imaging findings, in the absence of clear progression. Death was defined as in-hospital mortality during the index admission. Untraceable referred to cases in which treatment response could not be reliably assessed due to transfer, discharge against medical advice, or poor adherence resulting in incomplete outcome documentation.

### Statistical analysis

A 2×2 contingency table was constructed to calculate sensitivity, specificity, and accuracy. Comparative analyses were performed using McNemar’s Chi-squared test with the R statistical software package (version 3.6.2). Graphical illustrations were created using Adobe Illustrator (version 2020). A *p*-value of less than 0.05 was considered to indicate a statistically significant difference.

## Results

### Patient characteristics

A total of 261 patients with suspected LRTIs were enrolled and provided consent for sample collection and clinical screening. The cohort comprised 146 males and 115 females, with a median age of 58 years. Baseline characteristics of the patients at the time of admission are summarized in **Table 1**. The most common comorbidities included hypertension (36.1%), diabetes (29.5%), and hepatopathy (28.7%). Of the total patients, 237 (90.8%) cases had underlying pulmonary disease. Additionally, 51 patients had a history of malignancy. For all patients, the average number of days of hospitalization was 39 days.

**Table 1:** Clinical characteristics of samples in present study (n = 261).

The study cohort	Number of patients, n (%)
Age, years	58.0 [45.0; 70.0]
Sex	
Female	115 (44.1%)
Male	146 (55.9%)
Underlying disease	
Hypertension	94(36.1%)
Diabetes	77(29.5%)
Coronary atherosclerotic heart disease	14(5.3%)
Hepatopathy	75(28.7%)
Nephropathy	21(8.1%)
Tumour	51(19.5%)
Respiratory disorders	237(90.8%)
Immunological diseases	28(10.7%)
Gastrointestinal disease	36(13.8%)
Length of hospital stay (days)	39

### mNGS assay performance

**Figure 2:**
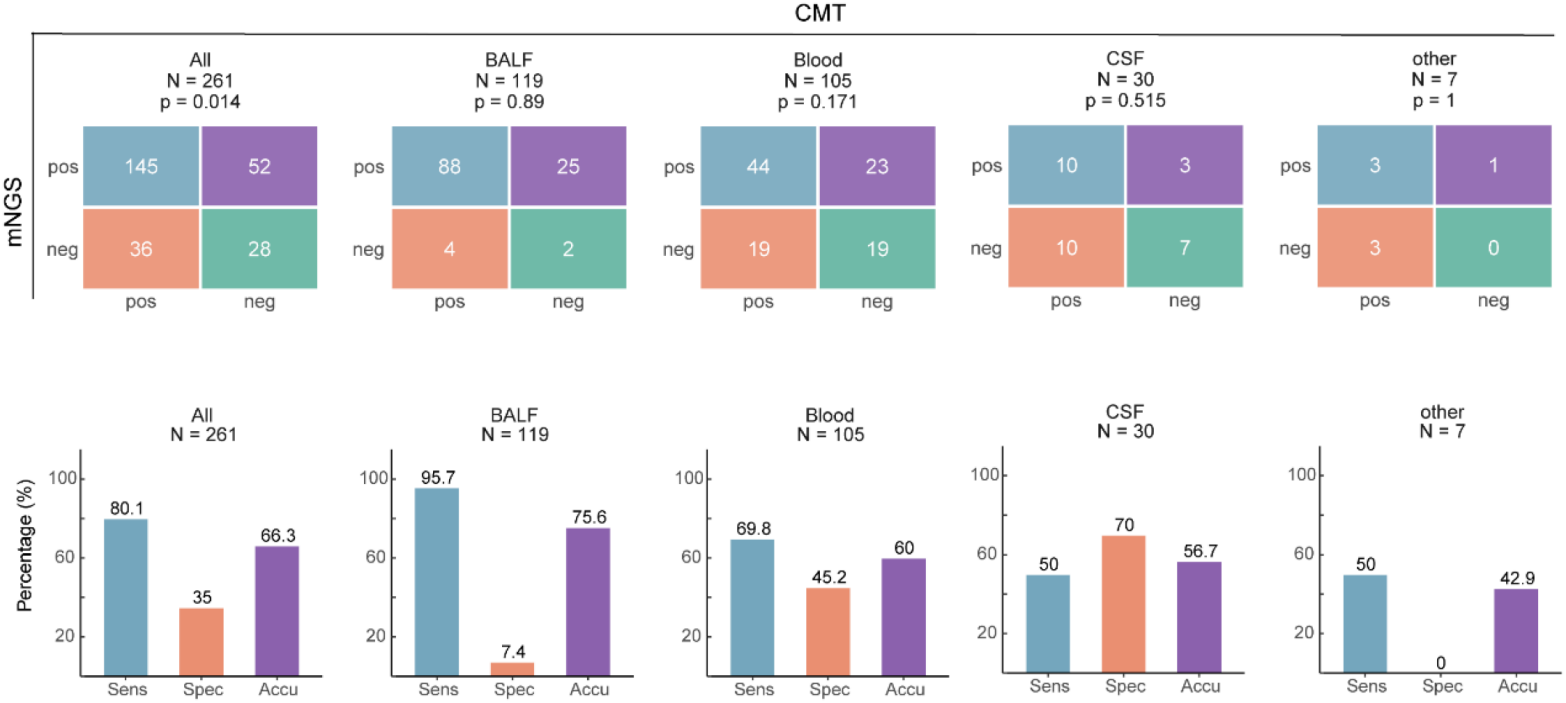
Diagnostic performance of mNGS across different specimen types.

Using CMT as the reference standard, the sensitivity and specificity of mNGS across all samples were 80.1% and 35%, respectively, with an overall accuracy of 66.3% (**Figure 2**). Among different sample types, mNGS demonstrated the highest sensitivity in BALF at 95.7%, followed by blood at 69.8%. In contrast, specificity was highest in CSF at 70%, but only 7.4% in BALF. The highest accuracy was observed in BALF samples, reaching 75.6%.

### Comparison of mNGS infection in 51 patients and CMT in species detection

**Figure 3:**
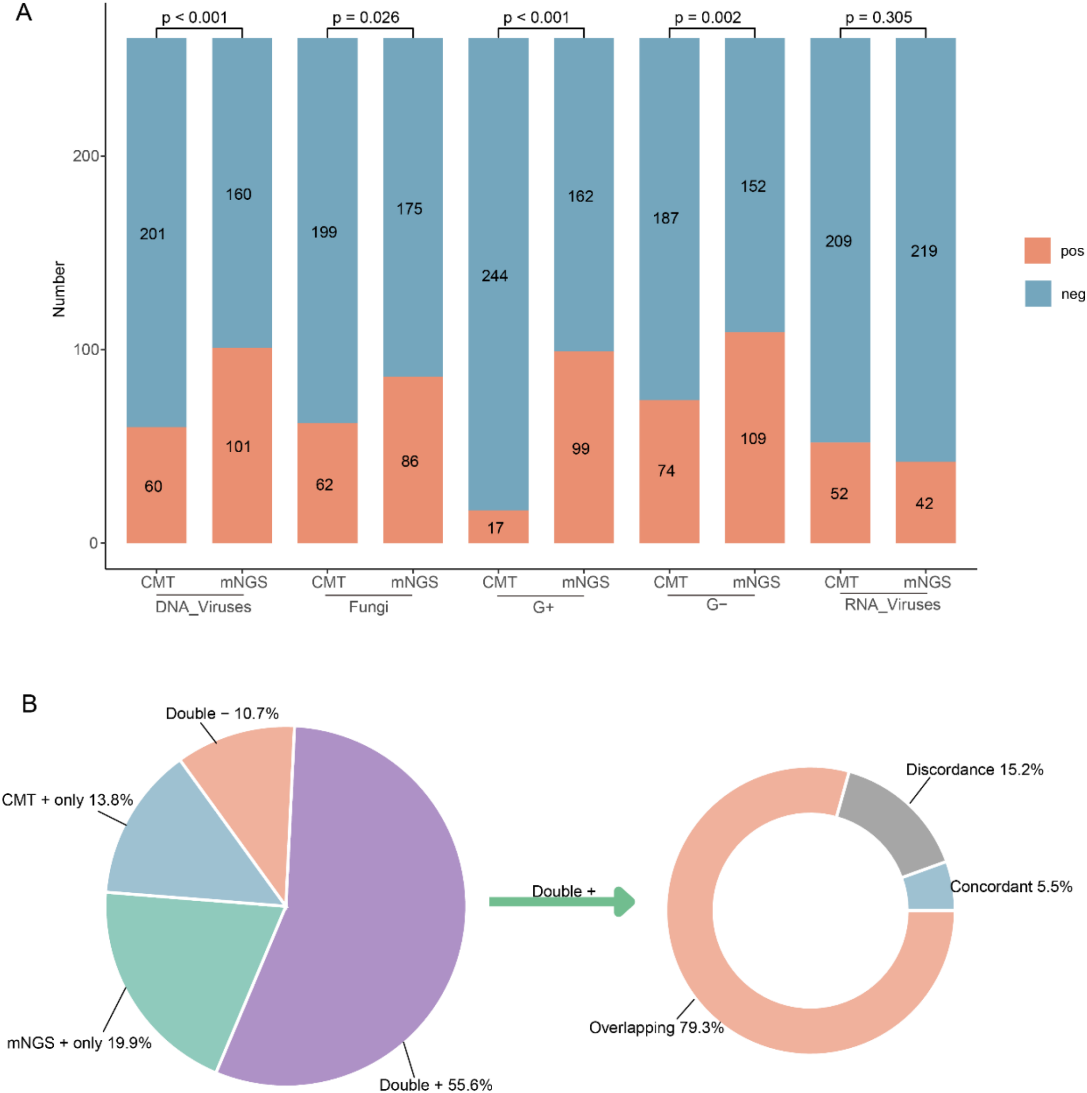
Comparison of pathogen detection between CMT and mNGS. **(A)** The positive rates on different species; **(B)** Consistency in species detection.

In addition to sample type, we compared the performance of mNGS and CMT in detecting bacterial, fungal, RNA viral, and DNA viral species (**Figure 3A**). Compared to CMT, mNGS demonstrated superior detection rates for Gram-positive bacteria (G+, 99 *vs.* 17), Gram-negative bacteria (G-, 109 *vs.* 74) and DNA viruses (102 *vs.* 62). These differences were statistically significant (*p* < 0.001). For fungi, the positive detection rate of mNGS was only slightly higher than that of CMT (33.0% *vs.* 23.8%, *p* = 0.026). In contrast, CMT showed a slight advantage over mNGS in detecting RNA viruses, although this difference was not statistically significant (*p* = 0.305).

Regarding the consistency of species detection, 55.6% of the samples were positive by both mNGS and CMT. However, mNGS uniquely detected pathogens in 52 samples, while CMT identified pathogens in 36 samples that were not detected by mNGS. Additionally, 28 samples were negative for pathogens by both methods. Among the 145 samples positive by both techniques, only 8 samples showed identical pathogen detection by mNGS and CMT. Overlapping pathogen detection was observed in 79.3% of the samples, while 22 samples exhibited discordant pathogen detection between the two methods (**Figure 3B**).

### Comparison of mNGS and CMT on pathogenic microorganisms

**Figure 4:**
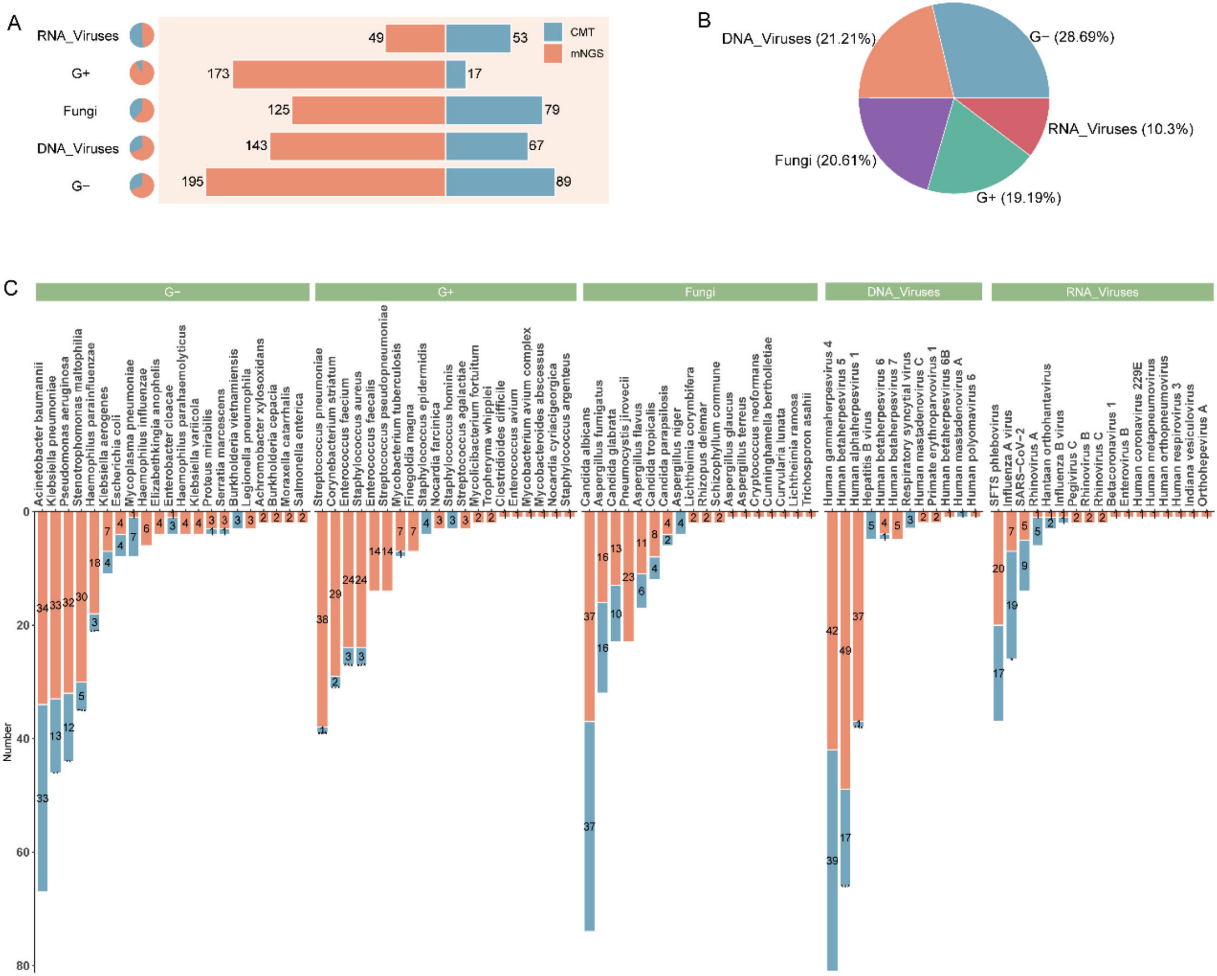
Pathogen detection profiles of mNGS and CMT. **(A)** detection counts across microbial categories; **(B)** distribution of microbial categories; and **(C)** species-level detection comparison.

We further analyzed the distribution of pathogenic microorganisms detected by mNGS and CMT. Two detection methods showed differences in the number of various microorganisms detected, particularly for G+ (173 vs. 17) and G- (195 vs. 89) (**Figure 4A**). Analysis of the proportion of different microorganisms showed that bacteria were the most frequently detected pathogens (47.88%), followed by DNA viruses (21.21%) (**Figure 4B**). For common bacterial pathogens such as *Klebsiella pneumoniae*, *Pseudomonas aeruginosa*, *Streptococcus pneumoniae*, and *Stenotrophomonas maltophilia*, significant differences in detection rates were observed between mNGS and CMT (*p* < 0.05). For fungal pathogens, the detection rates of *Aspergillus fumigatus* and *Candida albicans* were consistent between the two methods. However, *Pneumocystis jirovecii* was detected exclusively by mNGS, likely due to the limitations of CMT in identifying this organism. Among viral pathogens, the most frequently detected DNA and RNA viruses were Human gammaherpesvirus 4 and SFTS phlebovirus, respectively, with both methods showing largely consistent detection results. However, for Influenza A virus, CMT demonstrated a significantly higher detection rate compared to mNGS (*p* < 0.05) (**Figure 4C**).

### Value of mNGS results for clinical diagnosis

**Figure 5:**
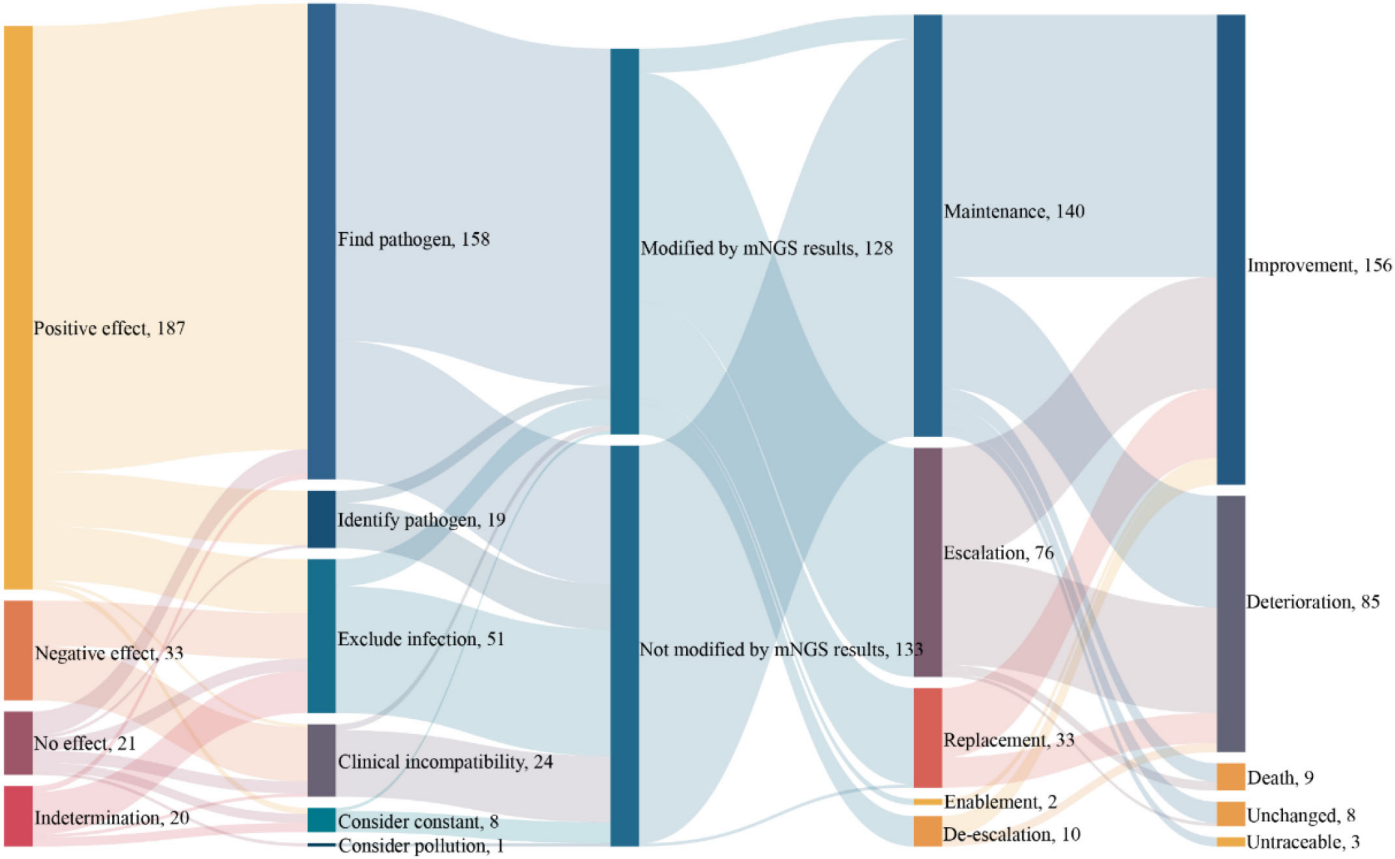
NGS-directed treatment modalities in ICU patients.

The impact of mNGS results on subsequent clinical management and outcomes was evaluated in this study (**Figure 5**). Among the patients, mNGS results had a positive influence on treatment decisions in 187 cases, a negative impact in 33 cases, and no or indeterminate impact in 21 and 20 cases, respectively. In terms of diagnostic significance, mNGS facilitated the identification of pathogenic microorganisms in 158 cases and helped rule out infection in 51 patients. Among the 128 patients whose treatment regimens were modified based on mNGS results, 59.4% underwent escalated therapy, 25.8% had changes in therapeutic agents, and 1.6% initiated a new treatment regimen. Regarding clinical outcomes, 58.6% of patients who adjusted their treatment regimens based on mNGS results demonstrated clinical improvement, while 37.5% experienced disease progression, 1.6% showed no change, and 2.3% died. These findings suggest the potential of mNGS to guide clinical decision-making and improve patient outcomes, particularly in cases where traditional methods fail to provide timely or informative diagnostic information.

### Exploration of microorganisms affecting patient outcomes

To investigate the relationship between microbial profiles and patient outcomes, patients were divided into survival (n = 198) and death (n = 63) groups. The pathogen spectrum was found to be richer in the survival group, particularly for bacteria and RNA viruses. Specifically, Huaiyangshan banyangvirus was detected exclusively in the survival group, along with coronaviruses, enteroviruses, and orthohantaviruses. In contrast, Human orthopneumovirus and Human metapneumovrus were detected only among non-survivors in this cohort. Among bacteria, Mycobacterium, Mycoplasma, and Chlamydia were more frequently detected in survivors, while *Cryptococcus neoformans* and *Rhizopus delemar* were detected only in the survival group (**Supplementary Figure 1**).

**Figure 6:**
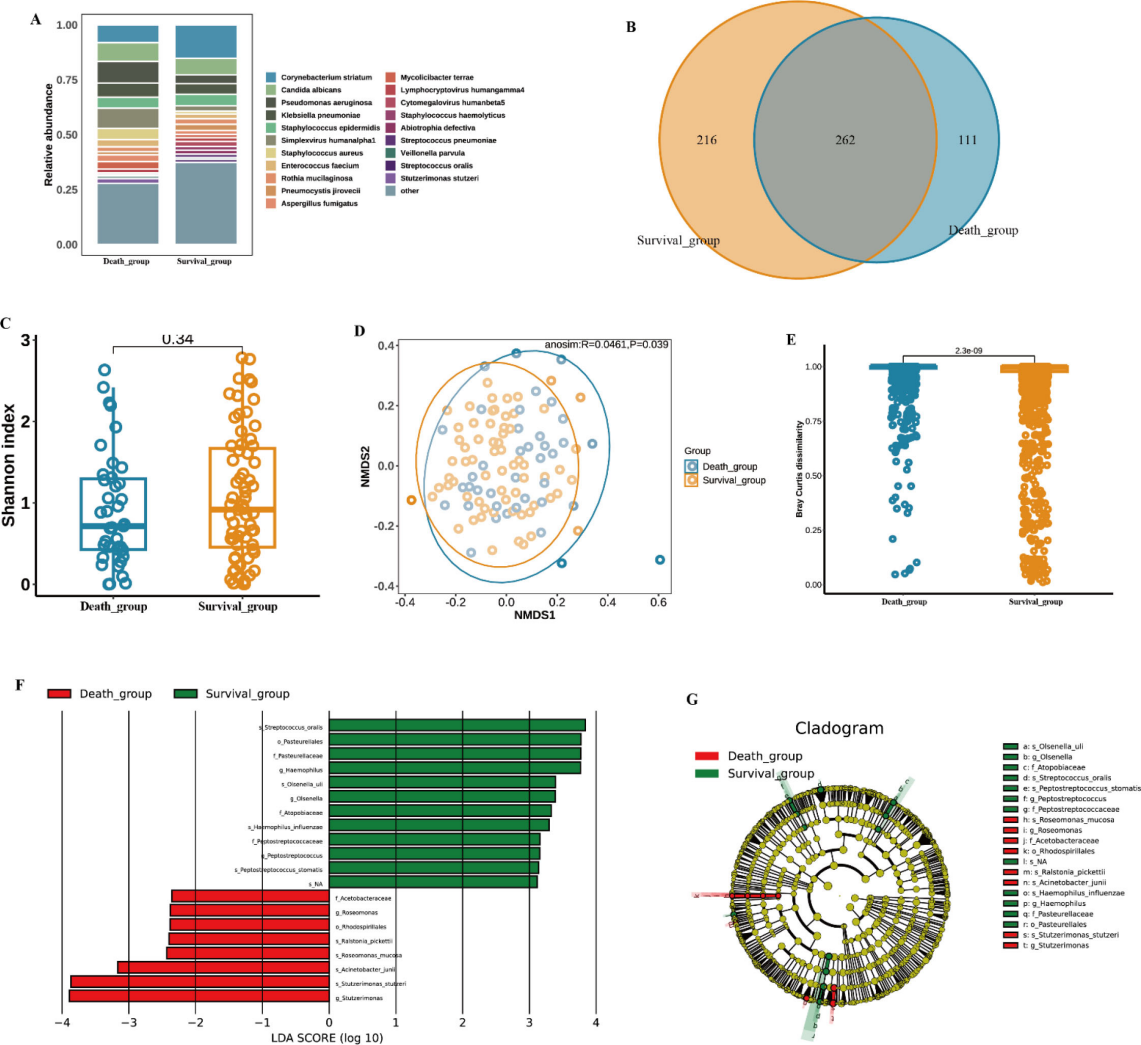
Microbiota characteristics associated with survival and death outcomes. **(A)** relative abundance of dominant species; **(B)** Venn diagram of shared and unique species; **(C)** Shannon index comparison; **(D)** β diversity comparison; **(E)** comparison of microbial community richness; **(F)** LDA effect size (LEfSe) analysis; and **(G)** cladogram representation of differential species.

In addition to pathogenic bacteria, we analyzed the bacterial flora of BALF from 119 patients (77 in the survival group and 42 in the death group). A total of 262 species were detected across both groups, with *Corynebacterium striatum* as the dominant bacterium in the survival group and *Pseudomonas aeruginosa* as the dominant bacterium in the death group (**Figures 6A, B**). However, no significant differences were observed between the two groups in terms of α-diversity or β-diversity (**Figures 6C–E**). LEfSe analysis identified 20 discriminatory biomarkers between the two groups, with 12 species in the survival group (*Streptococcus oralis*, *Pasteurellales*, *Pasteurellaceae*, *Haemophilus*, and *Olsenella uli*, etc.) and 8 species in the death group (*Stutzerimonas*, *Stutzerimonas stutzeri*, *Acinetobacter junii*, *Roseomonas mucosa* and so on) (**Figures 6F, G**).

### Construction of a predictive model for patient outcomes

**Figure 7:**
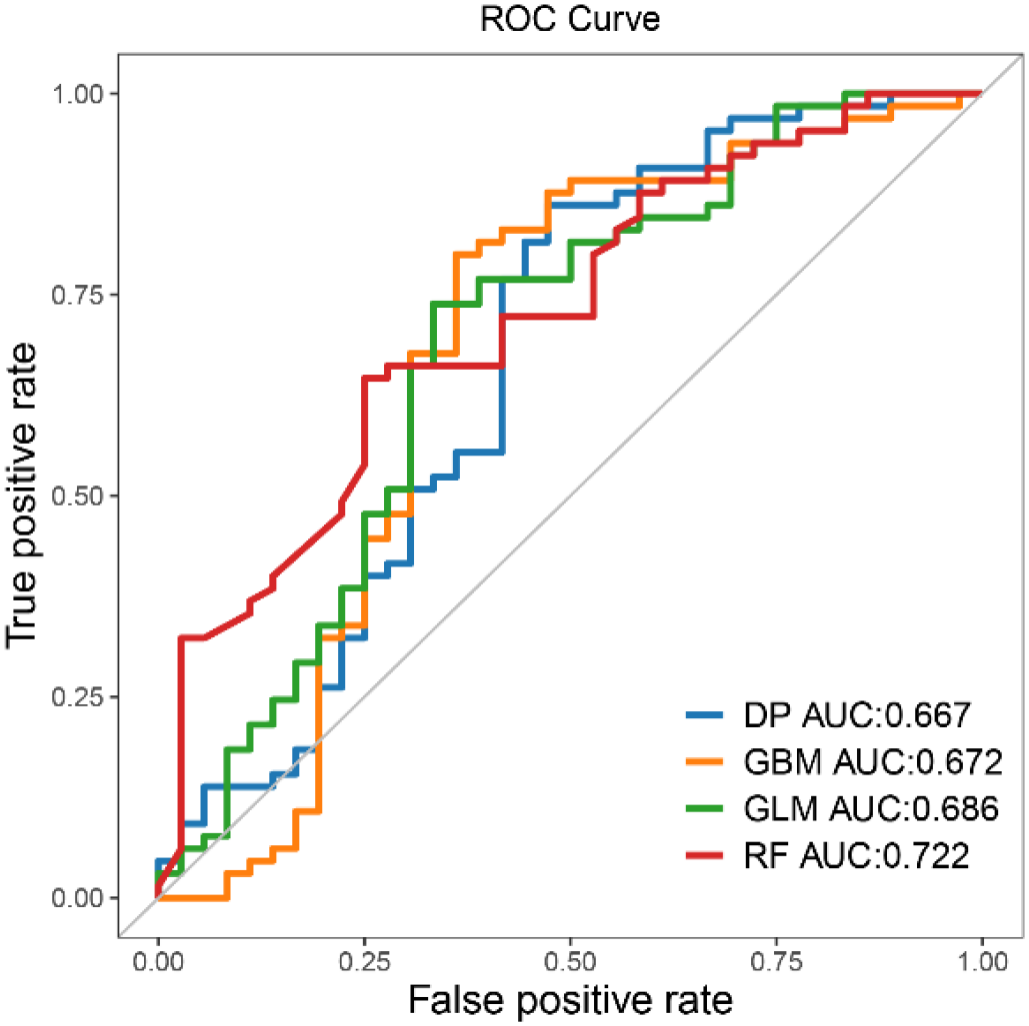
Receiver operating characteristic (ROC) curves for predicting patient survival and death outcomes using different machine learning models.

To identify the most reliable predictor of patient survival outcomes, we trained and compared four machine-learning classifiers built on differentially abundant species. Classification performance was evaluated with receiver operating characteristic (ROC) curve analyses (**Figure 7**; **Supplementary Table 1**). The random-forest (RF) model attained the highest area under the curve (AUC = 0.722), outperforming the generalize linear model (GLM, 0.686), gradient-boosting machine (GBM, 0.672) and deep learning (DP, 0.667) algorithms. These results indicated that RF model provides the strongest discriminative capacity for distinguishing survival from mortality, whereas the remaining models exhibit moderate but still appreciable predictive value.

## Discussion

In this study, we retrospectively analyzed the diagnosis of lower respiratory tract infection pathogenesis in critically ill ICU patients. The results demonstrated that mNGS exhibited high sensitivity for pathogen detection (80.1% overall, 95.7% in BALF samples) but low specificity (35% overall, only 7.4% in BALF samples). This is consistent with previous studies (Lv et al., 2024), indicating that the high sensitivity of mNGS can significantly enhance pathogen detection, particularly in cases of mixed infection (Hu et al., 2024). However, the low specificity could be attributed to the fact that traditional testing methods have more negative results, leading to low consistency when used as a reference standard for performance analysis. Therefore, in clinical practice, mNGS results should be interpreted in conjunction with the patient’s clinical manifestations and other test results for a comprehensive assessment, and clinicians should avoid over-reliance on a single technology.

Moreover, there were differences in detection efficacy between sample types. In BALF samples, mNGS exhibited significantly higher sensitivity for pathogen detection compared to blood and CSF samples, which may be related to the higher pathogen load in BALF in lower respiratory tract infections. However, the concordance between mNGS and CMT in blood samples was low (*p* = 0.171), indicating that pathogens of bloodstream infections may be difficult to capture due to low load or intermittent release (Holmes et al., 2025). Future studies need to further optimize the pretreatment process or sequencing depth of blood samples to improve their diagnostic value. For different species, mNGS was more advantageous in the detection of bacteria and DNA viruses, but not in fungi, which is also consistent with the results of existing studies (Fang et al., 2020; He et al., 2022b). This may be related to the need for further optimization of fungal cell wall lysis techniques to release more fungal nucleic acids for detection (Rodríguez et al., 2019). In addition, mNGS and CMT have unique strengths and limitations in detecting G+ and G- bacteria. mNGS offers broad coverage and can detect fastidious bacteria but may be confounded by background nucleic acids in clinical samples (Sun et al., 2025). In contrast, CMT is simpler, more cost-effective, and effective for common culturable bacteria but struggles with fastidious and anaerobic species (Liu et al., 2025). It is also necessary to emphasize that published studies have shown that mNGS can typically deliver results within approximately 24 h from specimen receipt, which is generally faster than culture-based workflows that often require at least three days for bacteria, around seven days for fungi, and several weeks for mycobacteria (Miao et al., 2018; Han et al., 2023; Gu et al., 2025). Nevertheless, the direct testing cost of mNGS is usually higher than that of conventional methods, and its overall cost-effectiveness likely depends on local laboratory workflows and downstream clinical impact.

In the analysis of pathogen spectra, differences in pathogen composition were observed between the survival and death groups; however, these comparisons may be influenced by the imbalance in group sizes. Bacteria (e.g., Mycobacterium, Mycoplasma) and RNA viruses (e.g., Huaiyangshan banyangvirus) were more abundant in the survival group, whereas human respiratory syncytial virus (HRSV), human metapneumovirus (HPV), and *Pseudomonas aeruginosa* were detected in the death group. *Pseudomonas aeruginosa* as the dominant bacterium in the death group may be associated with its strong virulence and drug resistance, as previous studies have shown that *Pseudomonas aeruginosa* is one of the life-threatening bacteria, accounting for 23% of all ICU-acquired infections, and has a high mortality rate in immunocompromised patients (Hafiz et al., 2023; Elfadadny et al., 2024). In contrast, the detection of Huaiyangshan banyangvirus in the survivor group may suggest that the virus is low virulence or the host immune response is effective (Song et al., 2018). In addition, the analysis of bacterial diversity showed a predominance of commensal bacteria such as *Corynebacterium striatum* in the survivor group, which may inhibit pathogen colonization through competition (Byrd et al., 2018), thus improving prognosis. These findings provide potential biomarkers for precision treatment of infectious diseases, but the mechanisms still need to be verified by functional experiments.

In this study, clinicians adjusted empirical anti-infective strategies for ICU patients based on mNGS results, which mainly included five types: escalation, de-escalation, maintenance, replacement and enablement. After the adjustment, 58.6% of the patients showed significant improvement in clinical symptoms, which indicates that mNGS is valuable in guiding the selection and use of antibiotics. A previous retrospective study reported an association between mNGS-informed antimicrobial adjustments and clinical management in ARDS due to severe pneumonia (Zhang et al., 2020), nevertheless, prospective studies are required to determine whether mNGS-guided strategies improve outcomes. Furthermore, disease progression still occurred in 37.5%, which may be related to the high mortality rate of ICU patients. Common causes of death include central nervous system failure, malignant tumors, and cardiovascular diseases (Mayr et al., 2006; Yan et al., 2024). In addition to this, clinical severity, age, and length of ICU stay are also risk factors for ICU mortality (Oliveira et al., 2024; Troisi et al., 2024; Papiol et al., 2025). This also suggests that clinical decision-making needs to weigh test results against individual patient factors such as immune status and underlying diseases.

Nevertheless, this study has several limitations. First, as a single-center retrospective analysis, it may be subject to selection bias, and the observed associations between mNGS-guided management and clinical outcomes require further confirmation in prospective studies. Second, the sample size for certain specimen types (e.g., CSF and other non-primary specimens) and across clinical subgroups was relatively limited, which may reduce statistical power and increase the risk of chance findings or false-positive results in differential microbiome/taxa comparisons. Third, we did not integrate host immune biomarkers or antimicrobial resistance information with the mNGS results, thereby limiting mechanistic interpretation of host-pathogen interactions. Future research should involve multicenter prospective studies that incorporate host transcriptomic or metabolomic data to comprehensively analyze infection-host interaction networks.

In conclusion, mNGS demonstrated high sensitivity in diagnosing lower respiratory tract infections. The association between the pathogen spectrum and prognosis offered new ideas for individualized treatment, and mNGS-guided antimicrobial strategies may help optimize therapy in ICU patients with suspected infection.

## Data Availability

All sequence reads were deposited into the Genome Sequence Archive in the National Genomics Data Center under the accession number PRJCA057924.

## Supplementary material

Supplementary Figure 1 Comparison of Pathogen Distribution in the Survivors and Deceased Groups.
