# Supplementary figures and images for: The value of metagenomic next-generation sequencing in lower respiratory tract infections among critically ill patients in the ICU
Source: Front Cell Infect Microbiol. 2026 Feb 25;16:1746117. doi: 10.3389/fcimb.2026.1746117 (PMC12976021; doi:10.3389/fcimb.2026.1746117)

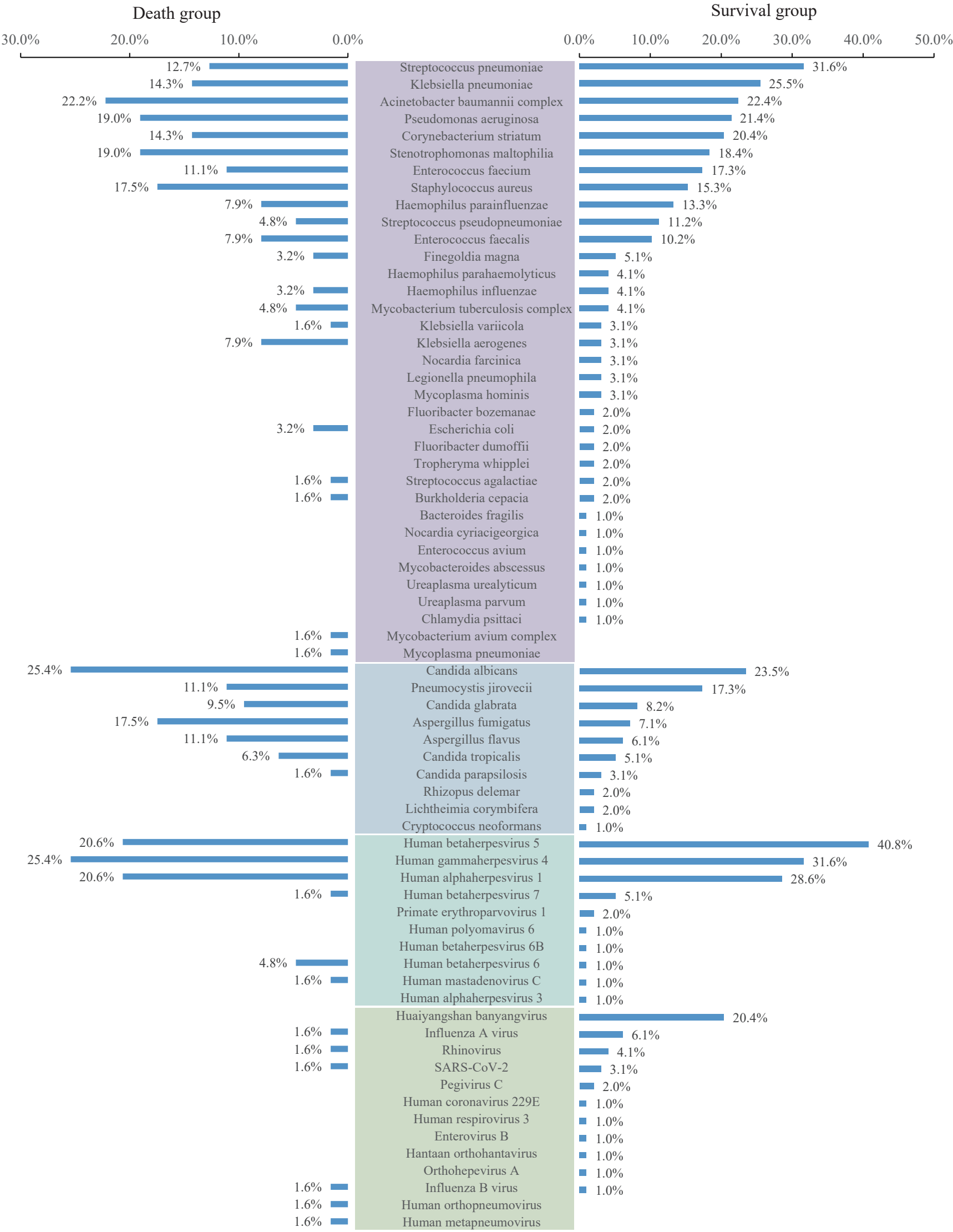

Supplement: Supplementary Figure 1 — Comparison of Pathogen Distribution in the Survivors and Deceased Groups. [file Image1.pdf]
