# Supplementary material for: The value of metagenomic next-generation sequencing in lower respiratory tract infections among critically ill patients in the ICU
Source: Front Cell Infect Microbiol. 2026 Feb 25;16:1746117. doi: 10.3389/fcimb.2026.1746117 (PMC12976021; doi:10.3389/fcimb.2026.1746117)
Supplement: Supplementary file 2 [file Table1.docx]

Supplemental Table 1 The performance of the prediction models

| Model | Threshold | Specificity | Sensitivity | CI_low | CI_high | Auc |
| --- | --- | --- | --- | --- | --- | --- |
| DP | 0.719 | 0.528 | 0.862 | 0.545 | 0.788 | 0.667 |
| GBM | 0.539 | 0.639 | 0.8 | 0.545 | 0.799 | 0.672 |
| GLM | 0.338 | 0.667 | 0.738 | 0.570 | 0.802 | 0.686 |
| RF | 0.323 | 0.75 | 0.646 | 0.619 | 0.825 | 0.722 |
